# Evaluation of the Effectiveness of Chitosan-Modified Bone Regeneration Materials: A Systematic Review

**DOI:** 10.3390/pharmaceutics17050665

**Published:** 2025-05-18

**Authors:** Tsvetalina Gerova-Vatsova, Stefan Peev, Ralitsa Yotsova, Varvara-Velika Rogova

**Affiliations:** 1 Department of Periodontology and Dental Implantology, Faculty of Dental Medicine, Medical University of Varna, 9002 Varna, Bulgaria; stefan.peev@mu-varna.bg; 2 Department of Oral Surgery, Faculty of Dental Medicine, Medical University of Varna, 9002 Varna, Bulgaria; ralitsa.yotsova@mu-varna.bg (R.Y.); varvara.rogova@mu-varna.bg (V.-V.R.)

**Keywords:** chitosan, bone graft, bone substitute, bone regeneration, biological properties

## Abstract

**Background/Objectives**: Today, regenerative therapy is routinely utilized in both medical and dental practices. Its outstanding results are due to the continuous development of technology and the invention of modern, more advanced biomaterials. The overarching idea in current regenerative therapy has shifted in the direction of the materials applied being osseointegrative, bioactive, responsive to stimuli from the body and actively promoting the overall regeneration of natural bone tissue. The aim is to determine whether chitosan is a material capable of improving the biological properties of different types of bone regeneration materials and, if so, which biological properties are affected. **Methods**: After going through the eligibility criteria, twenty articles, with a total of seventeen in vitro studies and six in vivo studies (some articles consisting of both), were included in this study. **Results**: The results presented colorimetric assays as the most commonly used methods investigating biological properties in in vitro studies, while in in vivo studies, researchers mainly rely on radiological and histological evaluation. After analyzing the data in this systematic review, it is clear that in vitro studies found a clear advantage of the results of chitosan-modified bone grafts in terms of bioactivity, osteogenic potential, biomineralization potential, biodegradability and antibacterial activity. In in vivo studies, chitosan-modified bone grafts stood out with better results in biocompatibility, osteogenic ability and biodegradability. **Conclusions**: In conclusion, it can be noted that chitosan-modified bone grafts have proven efficacy and the influence of chitosan is evidently favorable in terms of biological properties.

## 1. Introduction

Today, regenerative therapy is an integral part of modern medicine and dentistry. Although this type of treatment has proven itself over the years with impressive results, bone reconstruction surgery continues to evolve every day [1].

The composition and structure of native bone tissue must be understood in order to successfully develop materials for bone tissue engineering. Additionally, biomimetic natural and/or synthetic materials (biomaterials) must be chosen carefully [2,3].

To encourage the restoration of bone structure and functionality, bone regeneration necessitates a series of biological events involving several cell types and intracellular and extracellular communication networks [4]. It is for this reason that modern regenerative therapies are focused on the discovery and development of smarter biomaterials that respond to different stimuli that determine viability, proliferation, adhesion, cell growth and stem cell differentiation [5,6].

Biomaterials can provide a bone-like microenvironment by imitating the structure of the natural extracellular matrix (ECM) [7]. In support of tissue regeneration, this setting facilitates the recruitment of stem cells and controls a number of cellular processes, such as adhesion, proliferation, migration, and differentiation [8]. These biomaterials can also improve the synergistic effects of cytokines that support bone regeneration [9].

**Figure 1:**
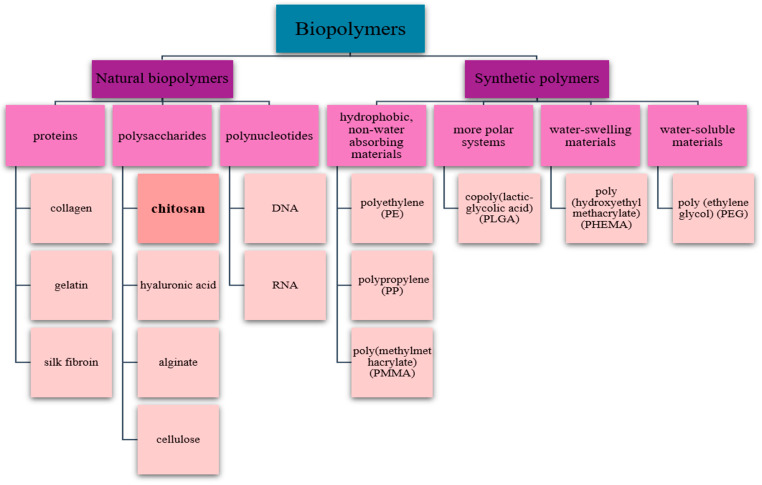
Brief classification of biopolymers.

Multiple repeating components known as monomers establish chemical bonds to form polymers. In order to create a polymer with certain qualities, choosing the right kind of monomer might be important [4]. The three primary categories of polymers that are frequently employed as biomaterials are synthetic non-biodegradable, synthetic biodegradable, and naturally produced polymers. Both in vitro and in vivo, these polymers can enhance the biological activity of cells by mimicking their ECM [9,10]. Figure 1 schematically presents a brief classification of biopolymers [11].

**Figure 2:**
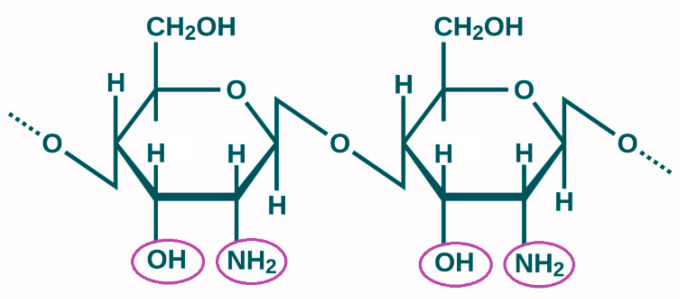
Chitosan chemical formula.

The focus of the present study is on the natural biopolymer—chitosan. It is well known that the safe polymers chitin and chitosan are important for tissue engineering and wound healing [10,12,13]. The alkaline deacetylation of chitin, which is mainly derived from shrimp shells and other crustaceans used in industry, yields chitosan, an aminopolysaccharide [12,14,15]. Chitosan, the second most common biopolymer in nature after cellulose, is made up of residues of 2-acetamido-2-deoxy-β-D glucopyranose and 2-amino-2-deoxy-β-D glucopyranose (b(1,4)-2-acetamido-D-glucose and b(1,4)-2-amino-D-glucose units). The primary functional groups in the chitosan structure that give it its unique features and limitless uses are -NH_2_ and -OH (Figure 2) [14].

For biomaterials to be successfully used in tissue engineering, their properties need to be well studied [16]. The characteristics that determine both the physical–mechanical and biological properties of chitosan are molecular weight, degree of deacetylation, crystallinity, total surface area and particle size [14,17]. Molecular mass is determined by the number of monomer units, and it is this mass that influences the viscosity and solubility of the material [12,14]. The degree of deacetylation represents the ratio between 2-acetamido-2-deoxy-β-D-glucopyranose and 2-amino-2-deoxy-β-D-glucopyranose [18,19]. The degree of acetylation during chitosan manufacture controls pH sensitivity and functionality, which are significantly impacted by the amine group in chitosan [19]. The proportion of a biopolymer’s crystalline to amorphous fractions is known as its crystallinity. There are three polymorphic forms of chitosan (α, β, and γ) that vary in their degree of crystallinity. The total surface area and particle size are determined by the source and extraction methodology of the chitosan. They determine the porosity of the material [14].

The qualities and scope of chitosan’s usage are determined by its features, which in turn determine its attributes [20]. It possesses outstanding gel-forming qualities and is soluble in acidic aqueous solutions but insoluble in water. It is non-toxic, biocompatible, and biodegradable, and it exhibits a variety of biological properties, including minimal immunogenicity and antibacterial activity [21,22]. All of these properties may have beneficial effects on osteoblast adhesion, proliferation and differentiation. This is the reason why chitosan is widely used in regenerative therapy today [22].

This systematic review’s objective is to figure out if chitosan is a material capable of improving the biological properties of different types of bone grafts, and if so, exactly which biological properties are affected. Special attention is also paid to the methods that have been used to investigate the biological properties in in vitro and in vivo studies. As far as we are aware, this is the first comprehensive evaluation that examines changes in the biological performance of chitosan-modified bone grafts.

## 2. Materials and Methods

Since this research is inclusively based on already published literature, it is not applicable due to the use of published literature.

### 2.1. Research Question

Based on the PCC framework, the following research question was created for this systematic review:

P (Population)—In vitro and in vivo (animals) studies.

C (Concept)—The effectiveness of chitosan-modified bone regeneration materials on bone regeneration.

C (Context)—Articles published between 2016 and 2025.

Research question: What is the effectiveness of chitosan-modified bone regeneration materials on bone regeneration (C), investigated through in vitro and in vivo (animals) studies (C) during the period from 2016 to 2025 (P)?

### 2.2. Eligibility Criteria

**Figure 3:**
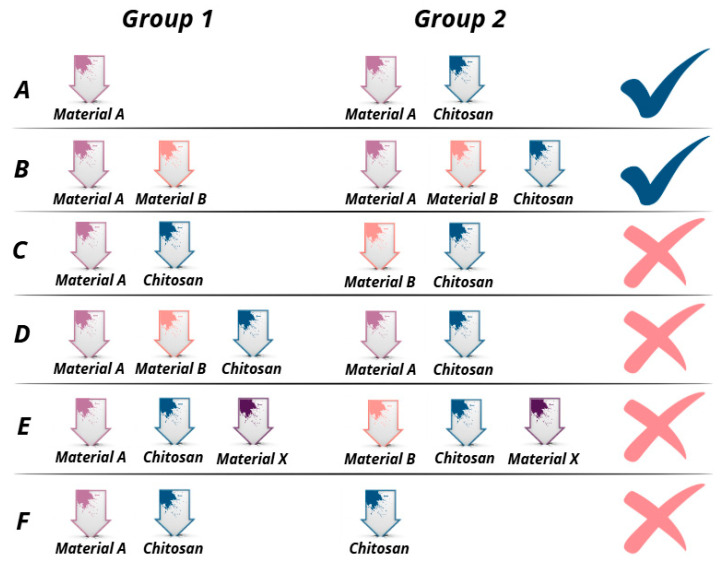
Eligibility criteria for inclusion and exclusion of studies.

Inclusion criteria:

- Research articles in English;
- Published in the period January 2016—January 2025;
- Studies including research on groups of bone grafts described in Figure 3A,B.

Exclusion criteria:

- Books, book chapters, reviews, case reports, case series, and abstracts;
- Articles published in 2014 and earlier;
- Articles written in non-English languages;
- Studies that do not evaluate the effectiveness of chitosan-modified bone regeneration materials;
- Studies investigating a variety of bone regeneration materials, but in which chitosan was included in all groups investigated (Figure 3C,D);
- Studies investigating the effectiveness of various bone grafts after the addition of chitosan in combination with another material (Figure 3E);
- Studies that examined chitosan-modified bone grafts, but the control group represented self-administered chitosan (Figure 3F).

To better illustrate the included articles in the present study that met the eligibility criteria, different variants of bone repair materials (group 1 and group 2) were presented and their results were compared with each other. Only studies (A and B) that compared the results between a given bone repair material or a given combination of bone repair materials with the same + added chitosan were eligible in the present study.

### 2.3. Information Sources

The Preferred Reporting Items for Systematic Reviews and Meta-Analyses (PRISMA) Statement standards are followed in this systematic review [23]. The International Platform of Registered Systematic Review and Meta-analysis Protocols (INPLASY) has been used to register this study with ID: INPLASY202540002.

On 21 February 2025, a thorough electronic search was carried out to find research publications in the Scopus, Web of Science, and PubMed databases.

### 2.4. Search Strategy

Only English-language, full-length research publications were included. An advanced Boolean search in the chosen databases was part of the search technique. The following keywords were utilized for the corresponding databases:

- Web of science—TS=(chitosan) AND (TS=(bone) AND (TS=(graft) OR TS=(substitute))) AND TS=(bone regeneration) AND TS=(biological properties)
- Scopus—(ALL (chitosan) AND ALL (“bone graft” OR “bone substitute”) AND ALL (“bone regeneration”) AND ALL (“biological properties”)) AND PUBYEAR > 2014 AND PUBYEAR < 2026 AND (LIMIT-TO (DOCTYPE, “ar”)) AND (LIMIT-TO (LANGUAGE, “English”))
- PubMed—(chitosan) AND (bone AND (graft OR substitute)) AND (bone regeneration) AND (biological properties)

Expanded search: ((“chitosan”[MeSH Terms] OR “chitosan”[All Fields] OR “chitosans”[All Fields] OR “chitosan s”[All Fields] OR “chitosane”[All Fields]) AND ((“bone and bones”[MeSH Terms] OR (“bone”[All Fields] AND “bones”[All Fields]) OR “bone and bones”[All Fields] OR “bone”[All Fields]) AND (“graft s”[All Fields] OR “grafted”[All Fields] OR “graftings”[All Fields] OR “transplantation”[MeSH Subheading] OR “transplantation”[All Fields] OR “grafting”[All Fields] OR “transplantation”[MeSH Terms] OR “grafts”[All Fields] OR “transplants”[MeSH Terms] OR “transplants”[All Fields] OR “graft”[All Fields] OR (“substitute”[All Fields] OR “substituted”[All Fields] OR “substitutent”[All Fields] OR “substitutents”[All Fields] OR “substitutes”[All Fields] OR “substituting”[All Fields] OR “substitution”[All Fields] OR “substitutional”[All Fields] OR “substitutions”[All Fields]))) AND (“bone regeneration”[MeSH Terms] OR (“bone”[All Fields] AND “regeneration”[All Fields]) OR “bone regeneration”[All Fields]) AND ((“biological products”[MeSH Terms] OR (“biological”[All Fields] AND “products”[All Fields]) OR “biological products”[All Fields] OR “biologic”[All Fields] OR “biologicals”[All Fields] OR “biological factors”[MeSH Terms] OR (“biological”[All Fields] AND “factors”[All Fields]) OR “biological factors”[All Fields] OR “biologics”[All Fields] OR “biologically”[All Fields] OR “biology”[MeSH Terms] OR “biology”[All Fields] OR “biological”[All Fields]) AND (“properties”[All Fields] OR “property”[All Fields]))) AND ((y_10[Filter]) AND (english[Filter])).

### 2.5. Study Selection and Data Collection Process

Abstracts and titles were reviewed and assessed for eligibility by two separate assessors (T.G. and R.Y.). Article titles, abstracts, authors, publication year and DOI-number were exported from the databases used (Web of Science, Scopus and PubMed) into an MS Excel spreadsheet. A review for available duplicate articles was performed and duplicates found were removed. All remaining full-text studies were screened for eligibility criteria in this systematic review. In order to perform an acute analysis of the data, studies meeting the criteria were divided into two tables, a table including in vitro studies and a table including in vivo studies. The table for in vitro studies includes information about Reference, Authors, Studied Materials, Study Method, Biological Properties. The table for in vivo studies includes information about Reference, Authors, Studied Materials, Model/Sample size, Studied period, Application, Study Methods, Biological Properties. The reviewers discussed and worked out disagreements until they came to an agreement.

### 2.6. Risk of Bias Assessment

The SYRCLE ROB tool for animal research was used to perform a quality evaluation for this study [24] and the QUIN assessing tool for in vitro studies [25]. The QUIN tool employs 12 criteria, with adequately specified criteria worth 2 points, inadequately specified criteria worth 1 point, not specified criteria worth 0 points, and not applicable criteria removed from the computation. The total score for a specific in vitro investigation is then calculated by adding the scores. The in vitro study was rated as high-, medium-, or low-risk based on the scores so obtained (>70% = low risk of bias, 50% to 70% = medium risk of bias, and <50% = high risk of bias). Two reviewers, T.G. and R.Y., independently assessed the risk of bias. The reviewers discussed and worked out disagreements until they came to an agreement.

## 3. Results

**Figure 4:**
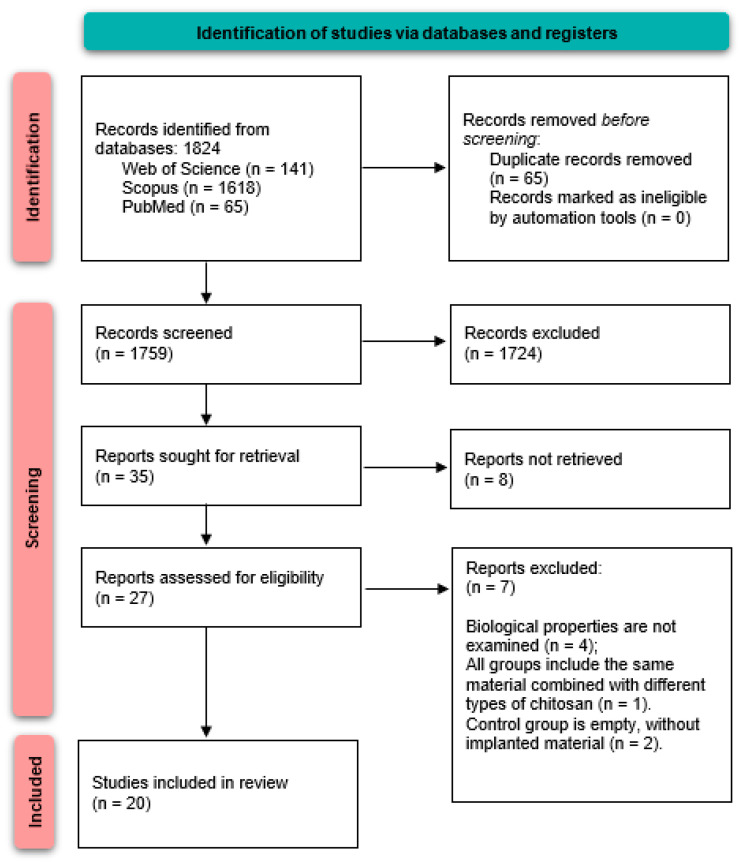
PRISMA flow diagram.

From the three different electronic databases, 1824 studies with possible topical relevance were found in the preliminary search. There were 1759 items left after 65 duplicate records were eliminated. Finally, 35 articles were assigned to the present systematic review. A PRISMA flow diagram showing the whole procedure of choosing pertinent articles is shown in Figure 4.

### 3.1. In Vitro Studies

In this systematic review, 17 in vitro studies were included (Table 1). These studies met all eligibility criteria and investigated at least two groups of bone grafts. One group included a given material or combination of materials, and the second group included the same material/combination but modified with chitosan. The features of each study are compiled in Table 1, which also includes the authors, references, studied materials, study methods, and biological properties. To synthesize and show the data, Microsoft Office Excel 2019 was utilized.

**Figure 5:**
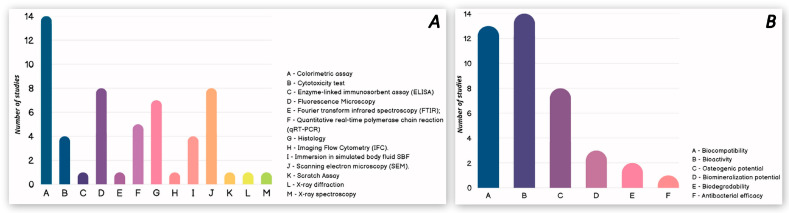
In vitro statistical summary. *(***A**). Methods used for investigating the biological properties. (**B**). Assessed biological properties—in vitro.

Among in vitro studies, colorimetric assays (CCK-8; MTT; MTS; XTT, etc.), followed by fluorescence microscopy, scanning electron microscopy (SEM) and histology are the most used methods investigating the biological properties that chitosan manages to influence after its use in combination with a bone regeneration material. Quantitative real-time polymerase chain reaction (qRT-PCR), cytotoxicity assays and immersion in simulated body fluid SBF were used to a lesser extent. The least used methods were enzyme-linked immunosorbent assay (ELISA), Fourier transform infrared spectroscopy (FTIR), imaging flow cytometry (IFC), scratch assay, X-ray diffraction and X-ray spectroscopy. Figure 5A presents each of the methods used and its degree of applicability.

The biological properties that have been evaluated among the in vitro studies selected in this systematic review are biocompatibility, bioactivity, osteogenic and biomineralization potential, biodegradability and antibacterial efficacy. Figure 5B presents all listed biological properties, arranged according to how many studies investigated them.

### 3.2. In Vivo Studies with Animals

Six in vivo studies were included in this systematic review (Table 2). These studies met all eligibility criteria and investigated a minimum of two groups of bone grafts. One group included a given material or combination of materials, and the second group included the same material/combination but modified with chitosan. References, authors, studied materials, model/sample size, studied period, application, study methods, and biological properties are all compiled in Table 2. To synthesize and show the data, Microsoft Office Excel 2019 was utilized.

**Figure 6:**
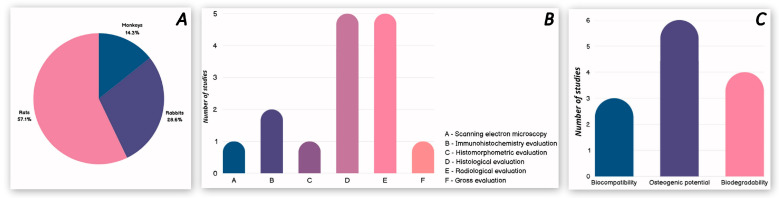
In vivo statistical summary. (**A**). Studied animal models. (**B**). Study methods used—in vivo. (**C**). Assessed biological properties—in vivo.

The in vivo studies that satisfied the requirements for eligibility and were selected for this systematic review used different animal models. Rats (n = 4) were the most often used model, followed by rabbits (n = 2) and monkeys (n = 1). The distribution of each model in our review is shown in Figure 6A.

Among in vivo (animal) studies, the most used methods investigating the biological properties that chitosan manages to influence after its use in combination with a bone regeneration material are histological and radiological evaluations, followed by immunohistochemistry evaluations. The least used methods are SEM, histomorphometric evaluation and gross evaluation. Figure 6B presents each of the used methods of examination and its degree of usability.

The biological properties that have been assessed among the in vivo studies included in this systematic review are biocompatibility, osteogenic potential and biodegradability. Figure 6C presents all listed biological properties, arranged according to how many studies investigated them.

### 3.3. Risk of Bias Assessment

**Figure 7:**
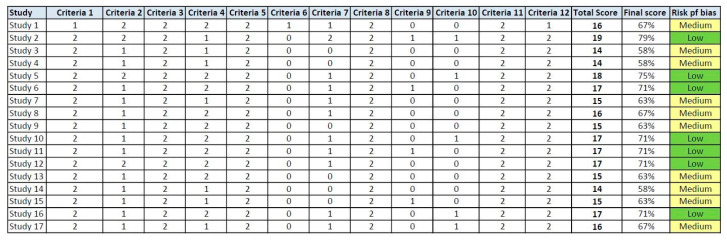
In vitro risk of bias assessment according to the QUIN assessment tool.

Among in vitro studies (Figure 7), the total risk of bias was evaluated as low/medium. Issues identified in certain studies primarily arose due to a lack of transparency regarding concealment, as well as insufficient data concerning the number of assessors or operators involved. Most studies presented clear aim, sample size/technique explanation, and nearly all of them specified clearly the performed statistical analysis and the results obtained. Overall, it was concluded that there is no high risk of bias in any of the seventeen studies and all of them shall be included in the systematic review.

**Figure 8:**
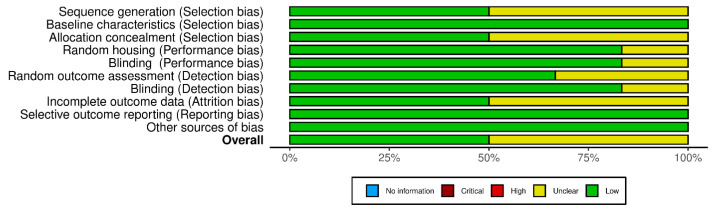
In vivo risk of bias assessment according to the QUIN assessment tool.

The findings for animal research (Figure 8) show minimal performance and reporting bias, as well as a low probability of selection bias for baseline characteristics. Though some domains present “Some concerns”, it is unlikely that these domains affect the achieved results. In summary, it was found that none of the studies show a significant risk of bias, so all shall be included in the systematic review.

## 4. Discussion

Over the past decade, there has been a consistent increase in the demand for the replacement and regeneration of bone defects resulting from chronic illnesses or traumatic incidents. Tissue engineering research has concentrated on biomedical applications to overcome these obstacles. This area focused on creating appropriate materials to improve bone integration and biological functionality [16]. The perfect bone regeneration material needs to provide both excellent biological and mechanical properties. To this end, it is increasingly common to combine different materials whose synergistic effect would overlap these requirements to some extent.

One promising new technique for regenerating and growing bones is tissue engineering (TE). The goal of TE is to create biomaterials that replicate the form and function of bones and promote natural bone healing [46,47].

Because of their advantageous antioxidant and anti-inflammatory qualities, biomaterials—including biopolymers—have attracted a lot of attention for their therapeutic potential in bone health and regeneration. Additionally, biopolymers are well-known for their beneficial qualities, which include structural resemblance to natural bones, biocompatibility, and biodegradability [48]. It is now evident that hybrid biomaterials may enhance a material’s biological qualities and have been effectively applied to implantology in medical settings [49].

The in vivo testing is a stage halfway between in vitro tests and human clinical trials, and the outcomes of the in vitro experiment greatly aid in the assessment of the direct cell reaction to the replacement biomaterial [50].

After analysis of the data from the in vitro studies in this systematic review, it is notable that seven of the seventeen studies used hydroxyapatite as the baseline study material. This can be explained by the fact that, because of its exceptional stability in the physiological environment and biological reactions, hydroxyapatite is the most widely employed phosphate substance in biomedical applications [51,52]. Ait Said et al. find that the two main materials being studied for biomedical applications are hydroxyapatite and chitosan biopolymer. As medication release mechanisms or bone replacements, both of these elements are crucial to the orthopedic industry. When used alone, hydroxyapatite is highly brittle, and chitosan has very little mechanical strength. Consequently, a blend of hydroxyapatite and chitosan polymers is employed, offering superior mechanical performance together with great biocompatibility and biomimetic potential [51].

Regarding the form of chitosan in the biomaterial matrix in the in vitro studies, it is clear that different concentrations of CS solution were used in 11 of the studies. It is also found that the most used concentrations of CS in the production of composite bone grafts are between 1 and 3 wt%. In their study of 2025, Guo et al. found that the higher the concentration of CS in the biomaterial matrix, the more pronounced the microporosity and, consequently, the more prominent the bioactivity of the cells [26]. In a study from 2023 by Wei et al., it was shown that at 3% CMCS/HA, biomineralization was more prominent compared to that at 1% CMCS/HA [32]. The data regarding the form and concentration of chitosan in the composites used in the in vivo studies is similar.

It became apparent that among the in vitro studies, a number of different research methods were applied, the aim of which was to reveal whether chitosan would affect the biological properties of a bone regeneration material. Once the data from the in vitro studies were analyzed, it was found that colorimetric assays (CCK-8; MTT; MTS; XTT, etc.), followed by fluorescence microscopy, SEM and histology are the most used methods. Among the assays, the most used colorimetric assays are MTT and CCK-8, which assess the biocompatibility of the materials under investigation. These methods are commonly applied in in vitro safety studies of innovative biomaterials, prior to their application in in vivo models [53,54]. Fluorescence microscopy, SEM and histology are the methods that are also used extensively among in vitro studies. However, through them, researchers mainly investigate the bioactivity of cells among the materials under study. These methods are commonly used among similar types of studies assessing bone regeneration by the application of a particular type of bone regeneration material [55,56,57].

In this systematic review, the main objective is to determine whether chitosan is a material capable of improving the biological properties of different types of bone grafts, and if so, which biological properties are affected. Among the 17 in vitro studies included, it can be seen that the biological properties being evaluated are biocompatibility, bioactivity, osteogenic potential, biomineralization potential, biodegradability and antibacterial efficacy. After analysis of the data, bioactivity was found to be the most studied biological property among those included in this systematic review of in vitro studies. In 14 out of 17 studies, bioactivity was investigated, and in absolutely all of them, it was found that chitosan improved the bioactive properties of the materials [26,27,29,30,32,33,34,35,37,38,39,40,41,42]. Biocompatibility has been investigated in 13 in vitro studies, but it is clear that no study found better results for groups with or without chitosan on this indicator [26,27,29,32,33,34,36,37,38,39,40,41,42]. Osteogenic potential was investigated in eight of the seventeen studies, and 100% found significantly improved results for the groups with chitosan included [26,30,33,34,38,39,41,42]. Regarding biomineralization potential, in two out of three studies [26,32], better results were reported in the group with chitosan included, while in the third group, no difference was found between the two samples for this indicator [28]. Biodegradability was examined in vitro in only two of the studies, but both found that the addition of chitosan to the material increased the time of degradation [30,32]. Moreover, the higher the chitosan content of the composite, the longer this time is [32]. A single study has examined antibacterial activity, demonstrating by LIVE/DEAD fluorescent staining that Fusobacterioum nucleatum and Porphyromonas gingivalis growth in planktonic cultures and biofilms may be inhibited by mHA/CS, compared to mHA [31].

Although in vitro research serves as the foundation for scientific investigations, it is insufficient to assess biological processes in live things [50]. Today, in vivo studies in animal models play an indispensable role in the testing of new bone regenerative materials. In 2015, Li et al. [50] concluded that the most commonly used small animal models in in vivo studies are rabbits and rodents. This is consistent with the findings obtained in the present systematic review. After data analysis, it was found that in different in vivo studies, the follow-up period after the surgical intervention varied from 1 to 12 weeks. This is most likely due to the different animal models used, their age, the different surgical site, etc. [58]. Furthermore, with respect to the application of the investigated materials, all included in vivo studies treated pre-existing bone defects. However, in one of the studies, before proceeding to bone defect regeneration in the midshaft of the ulna (rabbits), a degradability and inflammatory response test of the investigated materials was performed after subcutaneous implantation in rats. A similar type of study with subcutaneous implantation has been performed in other studies, but the materials studied did not contain chitosan [59,60].

After detailed examination and analysis of the information in the in vivo studies that met the eligibility criteria in this systematic review, it is clear that the most used methods investigating the biological properties that chitosan manages to influence after its use in combination with a bone regeneration material are histological and radiological evaluations. Through radiological methods, the volume of bone defect filling and bone density can be determined, which is key to demonstrating bone regeneration over a period of time [61]. In order to concretize the results and to comprehensively analyze the recovery processes, it is very common for researchers in these types of studies to consult histological studies [55,62].

In 2024, Lopes et al. showed that chitosan is a natural biopolymer that is widely used in tissue engineering due to its many advantages—biocompatibility, non-toxicity, antimicrobial properties and biodegradability [63]. There are a number of other studies in the literature that reflect the advantages of chitosan as a regenerative material [10,64,65]. In consensus with these claims, the results of the in vivo studies in our systematic review highlight three biological properties that chitosan manages to affect once added to a bone regeneration material—biocompatibility, osteogenic ability and biodegradability. In one study, for instance, it was evident that the CS/HA group’s results showed a larger synthesis of BMP-2 and a faster decline in proinflammatory cytokine (IL-1) production than HA [44]. In four other studies, it was clear that osteogenic potential was superior and accelerated over time in the groups with chitosan, compared with the counterparts without the presence of chitosan. This suggests that chitosan has an effect on the “osteogenic ability” parameter [26,41,42,45]. Interestingly, two of the studies reported a prolonged biodegradability period in the chitosan groups [42,43], but the study by Liu et al. in 2021 showed that CS/BCP degraded more rapidly than BCP [41]. Given the discrepancies obtained in the results on biodegradability, it would be good to look further in this direction in future studies.

It should be emphasized that in none of the in vitro and in vivo studies was chitosan found to negatively affect the results compared to those in the groups without chitosan included. This means that the indicated effectiveness of chitosan-modified bone regeneration materials and its suggested positive influence on them in terms of their biological properties can be noted as a key result of this systematic review.

## 5. Conclusions

The need for continuous development and discovery of new, better biomaterials is the basis for the idea of modifying already proven bone regeneration materials with chitosan. The reason for this lies in its time-proven biological properties.

In the present systematic review, it is clear that chitosan improves the biological properties of bone regenerative materials modified with it. In vitro studies found a clear advantage of the results of chitosan-modified bone grafts in terms of bioactivity, osteogen-ic potential, biomineralization potential, biodegradability and antibacterial activity. On the other hand, in vivo studies highlighted three biological properties that chitosan was able to influence once added to a bone regeneration material—biocompatibility, osteogenic ability and biodegradability. All of the above confirms the high regenerative potential of this type of composite material and fully justifies the idea of future research directed in this direction.

Among other things, this systematic review pays special attention to the methods used in in vitro and in vivo studies to demonstrate the effectiveness of the investigated materials in terms of biological properties.

## Figures and Tables

**Table 1:** In vitro studies included in this review.

Refs.	Authors	Studied Materials	Concentrations and Form of Chitosan	Study Methods	Biological Properties
[26]	Guo et al. (2025)	PLA-P ^1^ 0.25% CS ^2^/PLA-P 1% CS/PLA-P 3% CS/PLA-P	CS microporous foams produced with 0.25 wt%, 1 wt%, and 3 wt%	Colorimetric assay; Fluorescence Microscopy; Histology; Immersion in simulated body fluid SBF.	Biocompatibility
					Bioactivity
					Osteogenic potential
					Biomineralization potential
[27]	Huang et al. (2020)	BHA ^3^ 8% CS-BHA	CS solution (8% *w*/*v*)—by dissolving 4 g of CS in 50 mL of 1% acetic acid solution	Colorimetric assay; Fluorescence Microscopy.	Biocompatibility
					Bioactivity
[28]	Gallota et al. (2023)	HA ^4^ CS/HA	CS powder (10 wt% with respect to HA)	Immersion in simulated body fluid SBF	Biomineralization potential
[29]	Koski et al. (2020)	HA CS/HA	CS solution—by dissolving CS in 0.1 M acetic acid and pipetted on top of the HA disks at initial concentrations of 10 μg	Colorimetric assay	Biocompatibility
					Bioactivity
[30]	Gong et al. (2020)	K-struvite 1% O-CMC ^5^ + K-struvite ^6^ 2.5% O-CMC + K-struvite 5% O-CMC + K-struvite	O-CMC powders (0, 1, 2.5 and 5 wt.% with respect to K-struvite powder phase)	Colorimetric assay; Fluorescence Microscopy; Quantitative real-time polymerase chain reaction (qRT-PCR); Scanning electron microscopy (SEM).	Bioactivity
					Osteogenic potential
					Biodegradability
[31]	Liao et al. (2020)	3 mg/mL mHA ^7^ 2.25 mg/mL mHA/CS 4.5 mg/mL mHA/CS	CS solution—by dissolving 1 g of CS in 50 mL of 2% acetic acid solution. Chitosan was mixed with mHA at a mass ratio of 1:2	Fluorescence Microscopy	Antibacterial efficacy
[32]	Wei et al. (2023)	HA 1% CMCS ^8^/HA 3% CMCS/HA	CMCS powders (0, 1, and 3 wt.% with respect to HA)	Colorimetric assay; Cytotoxicity test; Fourier transform infrared spectroscopy (FTIR); Immersion in simulated body fluid SBF; Scanning electron microscopy (SEM).	Biocompatibility
					Bioactivity
					Biomineralization potential
					Biodegradability
[33]	Yang et al. (2022)	HA CS/HA	Not stated	Colorimetric assay; Histology; Imaging Flow Cytometry (IFC).	Biocompatibility
					Bioactivity
					Osteogenic potential
[34]	Najafabadi et al. (2024)	PMA ^9^ PMA/CS ^10^	CS solution—by dissolving 2 wt% of the Cs in acetic acid solution	Colorimetric assay; Histology; Immersion in simulated body fluid SBF; Scanning electron microscopy (SEM); X-ray diffraction; X-ray spectroscopy.	Biocompatibility
					Bioactivity
					Osteogenic potential
[35]	Lv et al. (2018)	SF ^11^ CS/SF	CS solution (1% *w*/*v*)—by dissolving CS in acetic acid solution	Cytotoxicity test; Enzyme-linked immunosorbent assay (ELISA); Scanning Electron Microscopy (SEM).	Bioactivity
[36]	Sampath & Krishnasamy (2024)	HA-GO ^12^ HA-GO-CS ^13^ Different concentrations (0.2, 0.4, 0.6, 0.8 и 1.0 μg/mL) of GO-HA and GO-HA-CS	CS solution—40 mL of 0.02 mg/mL chitosan was dispersed in 1% acetic acid	Colorimetric assay	Biocompatibility
[37]	Yildizbakan et al. (2024)	Fe^3+^-DCPD ^14^ Fe^3+^-DCPD: CS (20:80) Fe^3+^-DCPD: CS (30:70) Fe^3+^-DCPD: CS (40:60) Fe^3+^-DCPD: CS (50:50)	CS solution with 3 wt%—by dissolving high-molecular-weight chitosan flakes in 2% acetic acid	Colorimetric assay	Biocompatibility
					Bioactivity
[38]	Wang et al. (2022)	CS/CMC ^15^/MMT ^16^ CS/CMC/MMT-CM ^17^	CS solution—by dissolving 400 mg CS in 20 mL 1% glacial acetic acid solution	Colorimetric assay; Fluorescence Microscopy; Histology; Quantitative real-time polymerase chain reaction (qRT-PCR) assay.	Biocompatibility
					Bioactivity
					Osteogenic potential
[39]	Amiryaghoubi et al. (2022)	PCLUU (3%) ^18^ PCLUU (3%)/CS (1.5%)	CS solution—CS (3% *w*/*v*) was prepared in 2% acetic acid	Colorimetric assay; Fluorescence Microscopy; Histology; Quantitative real-time polymerase chain reaction (qRT-PCR) assay; Scanning electron microscopy (SEM).	Biocompatibility
					Bioactivity
					Osteogenic potential
[40]	Skubis-Sikora et al. (2024)	PCL ^19^ PCL/CS ^20^	Not stated	Colorimetric assay; Cytotoxicity test; Fluorescence Microscopy; Scanning electron microscopy (SEM); Scratch Assay.	Biocompatibility
					Bioactivity
[41]	Liu et al. (2021)	BCP ^21^ CS/BCP	CS solution with 2 wt%—80 mg CS was dissolved in 0.1 M acetic acid	Colorimetric assay; Fluorescence Microscopy; Histology; Scanning Electron Microscopy (SEM); Quantitative real-time polymerase chain reaction (qRT-PCR).	Biocompatibility
					Bioactivity
					Osteogenic potential
[42]	Zhou et al. (2024)	CFBB ^22^ CFBB/CS CFBB/Zn^2+^ CFBB/Zn^2+^/CS	CS solution—10 g/L solution of CS-acetic acid	Colorimetric assay; Cytotoxicity test; Histology; Scanning Electron Microscopy (SEM); Quantitative real-time polymerase chain reaction (qRT-PCR) assay.	Biocompatibility
					Bioactivity
					Osteogenic potential
					Biodegradability

^1^ PLA-P—polylactic acid-pearl; ^2^ CS—chitosan; ^3^ BHA—bovine-derived hydroxyapatite; ^4^ HA—hydroxyapatite; ^5^ O-CMC—oxygen-carboxymethyl chitosan; ^6^ K-struvite—magnesium potassium phosphate cement; ^7^ mHA—mesoporous hydroxyapatite; ^8^ CMCS—carboxymethyl chitosan; ^9^ PMA—3D-printed scaffold from polycaprolactone (PCL), magnetic mesoporous bioactive glass (MMBG) and alumina nanowire (Al2O3, 9 Optimal percentage 5%); ^10^ PMA/CS—PMA, coated with chitosan; ^11^ SF—silk fibroin; ^12^ HA-GO—hydroxyapatite–graphene oxide; ^13^ HA-GO-CS—hydroxyapatite–graphene oxide–chitosan; ^14^ Fe^3+^-DCPD—iron (III) nitrate-doped dicalcium phosphate dihydrate; ^15^ CMC—carboxymethyl cellulose; ^16^ MMT—montmorillonite; ^17^ CM—CS microsphere; ^18^ PCLUU—polycaprolactone-based polyurethane urea; ^19^ PCL—polycaprolactone; ^20^ PCL/CS—polycaprolactone/chitosan; ^21^ BCP—biphasic calcium phosphate; ^22^ CFBB—β-TCP-based calcined natural fetal bovine bone; Zn^2+^—zinc ions.

**Table 2:** In vivo studies included in this review.

Refs.	Authors	Studied Materials	Concentrations and Form of Chitosan	Model, Sample Size	Studied Period	Application	Study Methods	Biological Properties
[26]	Guo et al. (2025)	PLA-P ^1^ 1% CS ^2^/PLA-P	CS microporous foams produced with 1 wt%	rats, X	8 and 12 weeks	Bone defect regeneration (skull)	Radiological evaluation	Osteogenic potential
[41]	Liu et al. (2021)	BCP ^3^ CS/BCP	CS solution with 2 wt%—80 mg CS was dissolved in 0.1 M acetic acid	rats, 4	2 and 4 weeks	Subcutaneous implantation (Degradability and inflammatory response of scaffolds)	Histological evaluation	Biocompatibility Biodegradability
[42]	Zhou et al. (2024)	CFBB ^4^ CFBB/CS CFBB/Zn^2+^ CFBB/Zn^2+^/CS	CS solution—10 g/L solution of CS-acetic acid	rabbits, 12	6 and 12 weeks	Bone defect regeneration (ulna, unilateral)	Radiological evaluation; Histological evaluation.	Osteogenic potential
				rabbits, 48	4, 8 and 12 weeks	Bone defect regeneration: (mandibula, bilaterally)	Radiological evaluation; Histological evaluation; Immunohistochemistry evaluation.	Osteogenic potential Biodegradability
[43]	Oryan et al. (2016)	Gel ^5^ CS/Gel	CS solution—CS 2% was dissolved in a 1% acetic acid	rats, 10	8 weeks	Bone defect regeneration (radius, bilaterally)	Radiological evaluation; Gross evaluation; Histological evaluation; Histomorphometric evaluation; Scanning electron microscopy.	Biocompatibility Osteogenic potential Biodegradability
[44]	Gani et al. (2022)	HA ^6^ CS/HA	Not stated	rats, 18	1, 2 and 3 weeks	Bone defect regeneration (femur, bilaterally)	Immunohistochemistry evaluation	Biocompatibility Osteogenic potential
[45]	Zhang et al. (2018)	BG ^7^ + BMSCs BG/CSn ^8^ + BMSCs ^9^	CS solution—200 μL chitosan solution and 20 μL saline	monkeys, 4	12 weeks	Bone defect regeneration (extracted first and second premolars in the 4 quadrants)	Radiological evaluation; Histological evaluation.	Osteogenic potential Biodegradability

^1^ PLA-P—polylactic acid-pearl; ^2^ CS—chitosan; ^3^ BCP—biphasic calcium phosphate; ^4^ CFBB—β-TCP-based calcined natural fetal bovine bone; ^5^ Gel—gelatin; ^6^ HA—hydroxyapatite; ^7^ BG—bioactive glass block; ^8^ BG/CSn—bioactive glass block/chitosan nanoparticles; ^9^ BMSCs—bone marrow mesenchymal stem cells.

## Data Availability

The article contains the unique contributions made in this study. The corresponding author can be contacted with any additional questions.

## Abbreviations

The following abbreviations are used in this manuscript:

ECM	Extracellular matrix
SEM	Scanning electron microscopy
qRT-PCR	Quantitative real-time polymerase chain reaction
ELISA	Enzyme-linked immunosorbent assay
FTIR	Fourier transform infrared spectroscopy
IFC	Imaging flow cytometry
TE	Tissue engineering
CS	Chitosan
HA	Hydroxyapatite
BMP-2	Bone morphogenetic protein 2
Il-1	Interleukin-1
BCP	Biphasic calcium phosphate
